# Proteomic and Antibody Profiles Reveal Antigenic Composition and Signatures of Bacterial Ghost Vaccine of *Brucella abortus* A19

**DOI:** 10.3389/fimmu.2022.874871

**Published:** 2022-04-22

**Authors:** Chuan-Yu He, Jiang-Hua Yang, Yin-Bo Ye, Hai-Long Zhao, Meng-Zhi Liu, Qi-Lin Yang, Bao-Shan Liu, Sun He, Ze-Liang Chen

**Affiliations:** ^1^ Key Laboratory of Livestock Infectious Diseases, Ministry of Education, Shenyang Agricultural University, Shenyang, China; ^2^ Technology Center, Tecon Biological Co., Ltd., Urumqi, China; ^3^ Key Laboratory of Zoonose Prevention and Control at Universities of Inner Mongolia Autonomous Region, Innovative Institute of Zoonoses, Inner Mongolia Minzu University, Tongliao, China; ^4^ National Medical Products Administration (NMPA) Key Laboratory for Quality Monitoring and Evaluation of Vaccines and Biological Products, Key Laboratory of Tropical Diseases Control, School of Public Health, Sun Yat-sen University, Guangzhou, China

**Keywords:** brucellosis, bacterial ghost, vaccine, comparative proteomics, antibody profile

## Abstract

Brucellosis is an important zoonotic disease that causes great economic losses. Vaccine immunisation is the main strategy for the prevention and control of brucellosis. Although live attenuated vaccines play important roles in the prevention of this disease, they also have several limitations, such as residual virulence and difficulty in the differentiation of immunisation and infection. We developed and evaluated a new bacterial ghost vaccine of *Brucella abortus* A19 by a new double inactivation method. The results showed that the bacterial ghost vaccine of *Brucella* represents a more safe and efficient vaccine for brucellosis. We further characterised the antigenic components and signatures of the vaccine candidate A19BG. Here, we utilised a mass spectrometry-based label-free relative quantitative proteomics approach to investigate the global proteomics changes in A19BGs compared to its parental A19. The proteomic analysis identified 2014 proteins, 1116 of which were differentially expressed compared with those in A19. The common immunological proteins of OMPs (Bcsp31, Omp25, Omp10, Omp19, Omp28, and Omp2a), HSPs (DnaK, GroS, and GroL), and SodC were enriched in the proteome of A19BG. By protein micro array-based antibody profiling, significant differences were observed between A19BG and A19 immune response, and a number of signature immunogenic proteins were identified. Two of these proteins, the BMEII0032 and BMEI0892 proteins were significantly different (P < 0.01) in distinguishing between A19 and A19BG immune sera and were identified as differential diagnostic antigens for the A19BG vaccine candidate. In conclusion, using comparative proteomics and antibody profiling, protein components and signature antigens were identified for the ghost vaccine candidate A19BG, which are valuable for further developing the vaccine and its monitoring assays.

## Introduction

Brucellosis is a zoonotic disease caused by strains in the genus *Brucella*, of which spread and distribute globally (1). *Brucella* spp. includes 6 classical species and 7 new identified species, *Brucella melitensis* (*B. melitensis*), *Brucella* (*B. abortus*) and *Brucella suis* (*B. suis*) are the most pathogenic to both humans and animals (2). Animals can be infected through the digestive tract, respiratory tract, conjunctiva, mating, and contacting with contaminated secretions/faeces and aborted fetuses. The infected female animals mainly manifest as infertility, abortion, stillbirth, weak fetus, endometritis and mastitis. The main symptoms of male animals are orchitis and epididymitis. *Brucella* spp. will spread rapidly across the herd, burdening the brucellosis elimination and causing huge economic losses to the animal husbandry industries (3). Humans brucellosis occurred *via* consuming contaminated animal products (meat and milk), contacting infected animals and occupational aerosol exposure, and performing fever, spontaneous abortus, arthritis, and spondylitis (4). Therefore, immunising susceptible herds with available vaccines is optimal to control animal and human brucellosis (5).

Live attenuated vaccines are widely used for animal brucellosis control. The current available vaccines include *B. abortus* strain 19 (S19), *B. abortus* RB51, *B. suis* S2 (S2) and *B. melitensis* Rev.1, whereas they have some drawbacks (6). Firstly, the available licensed vaccines were all attenuated strains and remain pathogenic to susceptible animals, e.g., S19 can induce abortus in pregnant animals, cause infection, and interfere with the serological diagnosis (7). Secondly, RB51 and Rev 1 are resistant to rifampicin and streptomycin, respectively, making it difficult to treat reinfection (8, 9). Thus, more effective and safer vaccines are urgently needed.

Bacterial ghost is an alternative to tackle the potential virulence of live attenuated vaccines. The bacterial content is released outside by gentle biological or chemical methods, and the remaining intact bacterial envelope named bacterial ghosts (BGs) can be used as a vaccine component (10). BGs retain the complete surface morphology, structure and antigenic components, which are important for an immune response (11). BGs can directly enhance the proliferation of CD4+T cells and induce Th1/Th2 response, indirectly trigger CD8+T cells and participate in TLR4 dependent/independent pathway (11). Thus, BGs are applied as a platform delivering antigens and DNAs, promoting cross-presentation and enhancing antigen-specific immune response, such as increasing the production of interferon-gamma (IFN-γ) induced by CD8+ T cells (12). Like the inactive vaccine, BG vaccines are safe, convenient, and cost-effective. Mice immunised with *B. suis* S2 (13) or 2308ΔgntR (14) BG vaccines could elicit pathogen-specific serum IgG antibody response and sustain splenic T cell response, induced IFN-γ and IL-4 response, indicating the two BG vaccines exhibited protection against *B. melitensis* and S2308.

The above-mentioned *B. suis* S2 and 2308 BG vaccines are derived from *B. suis* and *B. abortus*, respectively. In addition, we had developed a ghost vaccine A19BG derived from *B. abortus* strain A19. Our results showed that A19BG provided similar protection in guinea pigs and cattle, safer than its parental strain (15). This study used comparative proteomics and protein microarray antibody profiling to gain insight into the mechanism and screen signature antigens for A19BG, which would be beneficial for brucellosis vaccine improvement and differential diagnosis.

## Materials and Methods

### Ethics Statement

Female Xinjiang Brown cattle (age 3–8 months) with no prior infection with *Brucella* were selected for analysis in this study and housed in an outdoor and restricted access isolation facility. All animal experiments were strictly performed in accordance with the Experimental Animal Regulation Ordinances (2017) formulated by the China National Science and Technology Commission. The protocol was approved by the Committee on Ethics and Welfare of Experimental Animals of Tecon biological Co., Ltd.

### Bacterial Strains and Plasmids

*B. abortus* A19 strain and recombinant plasmid pBBR1MCS-2E containing the E-lysis gene and thermosensitive element λpR-cI857 were constructed and preserved in Tecon Biological Co., Ltd (Urumqi, China) (15).

### A19BG Preparation and Collection

The method for constructing A19BG was described in detail in another work (15). In brief, the fragment containing the temperature-sensitive regulation system λpR-cI857 and E-gene lysis was amplified from plasmid pBV220::E. The polymerase chain reaction products were cloned into pBBR1MCS-2 to generate the lysis plasmid pBBR1MCS-E (16, 17). The recombinant plasmid pBBR1MCS-2E containing the E-lysis gene and the thermosensitive element *λpR-cI857* was electroplated into *B. abortus* A19 competent cells under the conditions of 200 Ω, 25 µF, and 1800 V. The electroporated bacteria were spread on TSA plates containing 100 µg/mL of kanamycin (Sigma, USA) and incubated at 28°C for 48 h.

A single positive colony was selected and cultured using the shake-culture technique in a 10 mL *Brucella* broth medium (BD, USA) containing 100 µg/ml kanamycin at 28°C, 150 rpm for 48 h. Then, 2 mL of the bacterial suspension was re-inoculated into the 200 mL *Brucella* broth medium containing kanamycin (100 μg/mL) and incubated up to the logarithmic growth period (OD_600_ = 0.6–0.8) at 28°C. Next, the culture temperature was elevated to 42°C and culturing was performed for 72h. The bacteria pellet was collected, washed three times with deionised water, and resuspended in 2.5 mL of deionised water, followed by the addition of 7.5 mL of lysis solution and autoclaving before being sent to PTM BioLab, Inc (Hangzhou, China).

### Protein Extraction and Sample Preparation

Protein samples were prepared as described previously with some modifications (18). Briefly, 1% protease inhibitor (Merck Millipore, Germany) was added to samples followed by ultrasonic lysis and centrifugation. The supernatant was transferred to a new centrifuge tube, and the protein concentration was measured with a BCA kit (Thermo Scientific, USA). An equal amount of each sample was taken for enzymatic hydrolysis, and the volume was adjusted to the same with the lysis solution. One volume of pre-cooled acetone was added following mixing, and then four volumes of pre-cooled acetone were added before precipitation at -20°C for two hours. The precipitate was collected after centrifuging at 4,500 g for 5 min and washing twice with pre-cooled acetone. TEAB was added to the pellet to a final concentration of 200 mM after drying and then ultrasonically dispersed. Trypsin was added at a ratio of 1:50 (protease: protein, m/m), and hydrolysed overnight. Dithiothreitol (DTT) was added to a final concentration of 5 mM and incubated at 56°C for 30 min. Next, iodoacetamide (IAA) was added to make a final concentration of 11 mM and incubated for 15 min at room temperature in the dark. The samples before and after lysis at 42°C were used as two control groups.

### LC-MS/MS Analysis

The peptides were separated by Ultra-High Performance Liquid system (Thermo Scientific, USA), injected into the nano-electrospray ionisation (NSI) ion source for ionisation, and then entered into the Orbitrap Exploris™ 480 mass spectrometer (Thermo Scientific, USA) for analysis. The ion source voltage was set to 2.3 kV, the FAIMS compensation voltage (CV) was set to -45V and -65V, and then peptide precursor ions and their secondary fragments were detected and analysed by high-resolution Orbitrap. The scanning range of the primary mass spectrum was set to 400-1200 m/z, and the scanning resolution was 60000; the fixed starting point of the scanning range of the secondary mass spectrum was 110 m/z, the secondary scanning resolution was set to 15000, and TurboTMT was set to Off. The data acquisition mode was cycled time-based data-dependent scanning (DDA); that is, the peptide precursor ions were selected according to the order of signal intensity from high to low within a cycle of 1.0 s, and then entered the HCD collision cell using 27% fragmentation. The energy was fragmented, and the second-stage mass spectrometry analysis was also carried out sequentially. In order to improve the effective utilisation of the mass spectrometer, the automatic gain control (AGC) was set to 100%, the signal threshold was set to 5E4 ions/s, the maximum injection time was set to Auto, and the dynamic rejection time of the tandem mass spectrometry scan was set to 20s to avoid repetitive scan of precursor ions.

### Database Searching

The raw data from the mass spectrometer were imported into the database search software Proteome Discoverer (v2.4.1.15) for retrieval. The dataset was *Brucella*_abortus_biovar_1_strain_9941_262698_*Brucella*_abortus_strain_2308_359391_PR_20210301_combine_20210508.fasta (6100 sequences). Anti-database was added to calculate the false positive rate (FDR) caused by random matching. The common pollution database was added to eliminate the influence of contaminating proteins in the medium. The restriction digestion method was set to Trypsin (Full). The number of missed cleavage sites was set to 2. The minimum length of the peptide was set to 6 amino acid residues. The maximum modification number of the peptide was set to 3. The mass error tolerance of the precursor ions and the secondary fragment ion was set to 10 ppm and 0.02 Da, respectively. Carbamidomethyl was specified as a fixed modification, while oxidation, acetyl (N-terminus), met-loss, and met-loss+acetyl were specified as variable modifications. The FDR for protein, peptide, and PSM identification was set to 1%.

### Bioinformatic Analysis

Gene Ontology (GO) annotation of proteins was based on three categories: molecular function, biological process, and cellular component (19). GO annotation was executed *via* eggnog-mapper software (v2.0) based on the eggnog database. Kyoto Encyclopaedia of Genes and Genomes (KEGG) database was employed to annotate the pathways in which differentially expressed proteins (DEPs) are involved (20). Clusters of Orthologous Groups (COG) database was used to assign the distribution of DEPs (21).

### Protein Microarray Antibody Profiles

The microarray was developed by using *in vitro* expression of a cloned recombinant expression vector and aldehyde-modified microarray with a fluorescently labelled (CY5) histidine antibody. Antibodies against IgG were labelled with CY5 and fluorescently labelled secondary antibodies were prepared; immune sera were reacted with the microarrays, followed by reactions with different concentrations of fluorescently labelled secondary antibodies, and the concentrations of primary and secondary antibodies were determined by reaction intensity analysis to establish the microarray detection method for the antibodies. Fifteen *Brucella* antibody-negative female Xinjiang Brown cattle (age 5–8 months) were randomly divided into three groups (n = 5 per group). In the A19 group, each animal was subcutaneously immunised with 6.0 × 10^10^ CFU of the A19 vaccine on the neck. In the A19BG group, the cattle were intramuscularly injected with 5.0 × 10^10^ BGs A19BG vaccine on the buttocks. Finally, the control group was injected with normal saline. The cattle were isolated and reared under the same conditions. The sera were selected at 28 days-post immunisation (14 days post-booster immunisation) and reacted with the proteomic microarray, while the levels of IgG antibodies in the serum was measured.

### Preparation of *Brucella* BMEII0032 and BMEI0892 Recombinant Protein

The open reading frames of BMEII0032 and BMEI0892 were amplified by PCR using the DNA from the A19 strain. The amplified DNA fragments were cloned into the pET-28a (Thermo Scientific, USA) vector and transformed in *E. coli* BL21 (DE3) cells (Transgen, Beijing). The transformed *E. coli* BL21 cells carrying the pET-28a-BMEII0032 and BMEI0892 plasmid were used for expression studies. Single colonies of transformed cells are incubated overnight at 37°C with continuous shaking at 200 rpm in a 5 ml LB broth medium containing kanamycin (100 µl/ml). 500 μl of culture material was removed and incubated in 200 ml LB broth. The cultures were grown to OD600 = 0.6–0.8at 37°C with vigorous shaking at 200 rpm. Isopropyl-β-D-thiogalactopyranoside (Invitrogen, USA) was added to a final concentration of 1 mM for the expression of BMEII0032 and BMEI0892 recombinant protein. Incubation was continued for 4 hours at 37°C with 200 rpm oscillation. The recombinant proteins were separated and analysed with SDS-PAGE (12%). The recombinant proteins, BMEII0032 and BMEI0892, was purified using an affinity chromatography Ni-NTA column (Cytiva, Sweden), protein folding with Pierce Protein Refolding Kit (Thermo scientific Number 89867, USA). Bradford method with bovine serum albumin (BSA) as a standard was used to assay protein concentration.

### Immunoreactivity of Recombinant *Brucella* BMEII0032 and BMEI0892 to Cattle Sera Using ELISA

Clinical sera from bovine immunised with A19 and A19BG respectively were analysed by indirect ELISA using recombinant BMEII0032 or BMEI0892 as antigens. Immunoassay plates (Corning 42592, USA) were coated with purified recombinant BMEII0032 and BMEI0892 proteins at a 1 µg/ml concentration in the carbonate coating solution and incubated overnight at 4°C. The wells were emptied and washed 3 times with phosphate-buffered saline-Tween 20 (PBST) and then closed with 10% rabbit serum (SBJ Bio, Nanjing, China) at 37°C for 2 hours. Fill the plate with 1/100 dilution of serum and incubate at 37°C for 1 hour. After 5 washes with PBST, the plates are incubated with HRP coupling at 37°C for 1 hour. After washing with PBST, a substrate solution containing TMB (3,3′,5,5′-tetra methyl benzidine, KPL, USA) was loaded into the wells of the plate and the plate was incubated in the dark for 5 minutes at room temperature before the reaction was terminated by the addition of a termination solution (Sera care Life Sciences KPL TMB Microwell Peroxidase). The absorbance was measured at 650 nm in an ELISA reader (Bio-Rad, USA). Each sample was run in duplicate. In addition, as a control for each serum, wells were left uncoated.

### Statistical Analysis

The data were analysed by one-way ANOVA using SPSS 20 (IBM, Armonk, NY, USA). The significant differences among treatments were tested based on the least significant difference (LSD) at P ≤ 0.01.

## Result

### Characterisation of A19 and A19BG Proteome

A total of 665,382 mass spectra were generated and 21,942 specific spectra were obtained from the original data with an FDR of 1.0%. Each protein contained at least one specific peptide and the number of proteins could be further quantified to 2,014 (**Figure S1A**, **Supplementary Table S1**). For comparative analysis, A19 lysed at 42°C was set as the experimental group (A19BG), and A19 cultured at 28°C was set as the control group (A19). With ratios of 1.50 and 0.67 as the cutoffs for differential up and downregulated expression, the numbers of DEPs that were significantly upregulated and down-regulated in A19BG group were 535 and 581, respectively (**Figure S1B**, **Supplementary Table S2**). Quality control results showed that most peptides were 7–20 amino acids in length (**Figure S1C**), which conformed to the general rules based on trypsin enzymatic hydrolysis and HCD fragmentation. Most proteins corresponded to more than two peptides (**Supplementary Table S1**) and were beneficial to increasing the quantitative results’ accuracy and credibility. The coverage of most proteins was below 20%, and the distribution of proteins above 10 kD was relatively uniform (**Figure S1D**), indicating that there was no obvious bias in the molecular weight of the proteins weighing more than 10 kD, and that proteins with a larger molecular weight (above 100 kD) were not lost due to poor solubility during the preparation process. The repeatability test of samples, including principal component analysis and Pearson’s correlation coefficient (**Figures S1E, F**), also showed a good repeatability.

### Functional Analysis of DEPs

**Figure 1:**
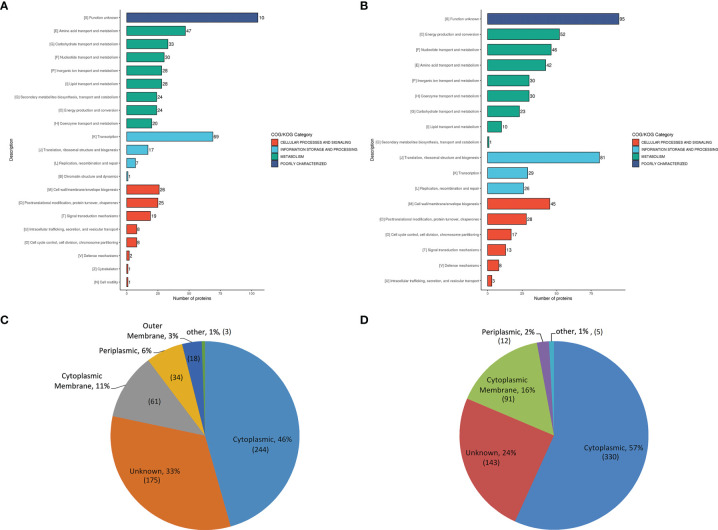
COG and subcellular location analysis of DEPs. **(A, B)**, the histogram displaying COG analysis of upregulated **(A)** and down-regulated **(B)** DEPs in A19BG. Letters displayed in the abscissa represent individual COGs with the numbers of proteins listed in brackets afterwards. **(C, D)**, Annotated classification of subcellular structures of upregulated **(C)** and down-regulated **(D)** DEPs in A19BG.

Most upregulated DEPs were related to the transportation and metabolism of multiple substances, and transcription (**Figure 1A** and **Supplementary Table S3**). The functional classification of most down-regulated DEPs included the transportation and metabolism of multiple substances, translation, structure and biosynthesis of ribosome, energy production and conversion, and cell wall/membrane/envelope biosynthesis (**Figure 1B** and **Supplementary Table S3**). The number of upregulated DEPs involved in material transportation and metabolism (210) was higher than that of down-regulated ones (182), indicating that the metabolic activity was increased, while energy production was decreased in the A19BG group. In addition, the upregulated DEPs were predominant in transcription function, but the down-regulated DEPs were predominant in the translation, ribosome structure and biosynthesis functions, indicating that only the upstream transcription might be carried out efficiently, while the downstream process of ribosomal translation might be silenced or even blocked in the protein synthesis of A19BG.

### Subcellular Localization Analysis of DEPs

The results showed that the down-regulated DEPs were mainly located in the cytoplasm (57%) followed by cytoplasmic membrane (16%) (**Figure 1D** and **Supplementary Table S4**). The number of down-regulated DEPs (421) in the cytoplasmic membrane and cytoplasm was higher than that of upregulated ones (305), and DEPs located in the outer membrane were all upregulated proteins (**Figure 1C** and **Supplementary Table S4**).

### Functional Enrichment and Cluster Analysis

**Figure 2:**
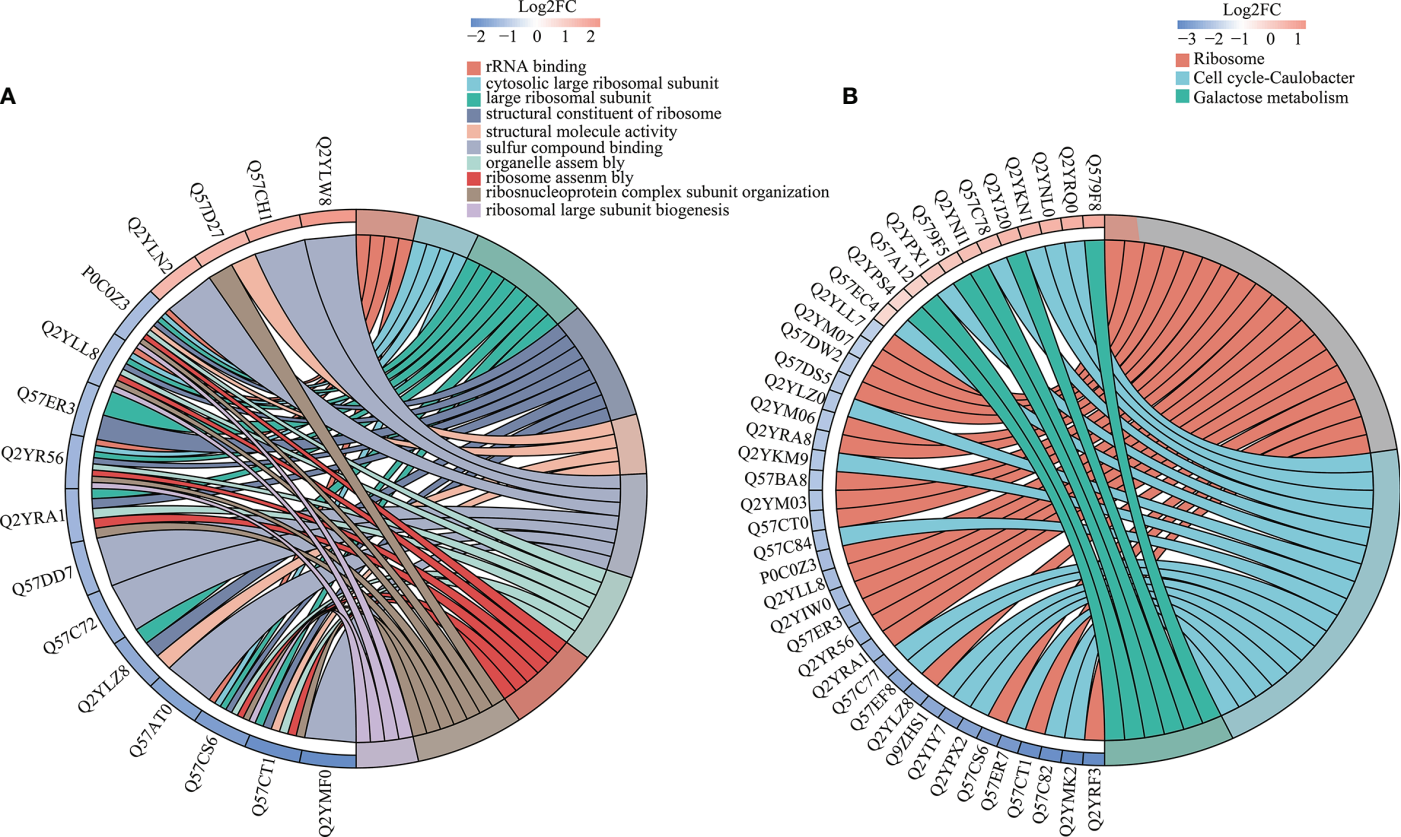
Differential GO and KEGG enrichment in A19 and A19BG. **(A)** GO differential protein function enrichment. **(B)** KEGG differential protein function enrichment.

GO enrichment was analysed based on biological process, molecular function and cellular component (**Supplementary Table S5**). The results showed that most upregulated DEPs after treatment were enriched in cell envelope and periplasmic space. After lysis, the ribosomal and intracytoplasmic metabolism-related proteins were down-regulated in A19BG, such as the ribosome, ribosomal subunit, structural constituent of ribosome, structural molecule activity, rRNA binding, organelle assembly and ribosome assembly, indicating that E protein cleavage inhibits A19 proliferation and leads to loss of cellular contents (**Figure 2A**). The analysis of KEGG functional enrichment showed that the DEPs were mainly concentrated in the ribosome, cell cycle-Caulobacter and galactose metabolism (**Figure 2B**).

**Figure 3:**
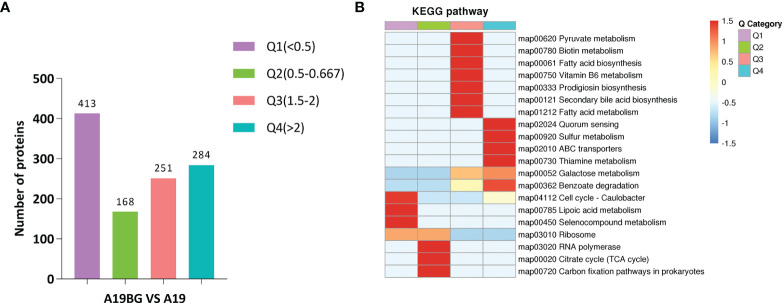
Cluster analysis of KEGG pathways. **(A)** Number of proteins with different differential expression multiples. **(B)** KEGG cluster analysis of the Q1-Q4 subgroups. According to the differential expression multiple, the DEPs were divided into four groups, called Q1 to Q4. The functions of interest in the different groups were clustered together using hierarchical clustering based on Fisher’s exact test p-values obtained from the enrichment analysis and plotted as a heatmap. the horizontal side of the heatmap represents the results of the enrichment test for the different groups and the vertical side is a description of the differentially expressed enrichment-related functions are depicted. The colour blocks corresponding to the descriptions of differentially expressed proteins and functions in different groups indicate the degree of enrichment. Red indicates strong enrichment, and blue indicates weak enrichment.

**Figure 4:**
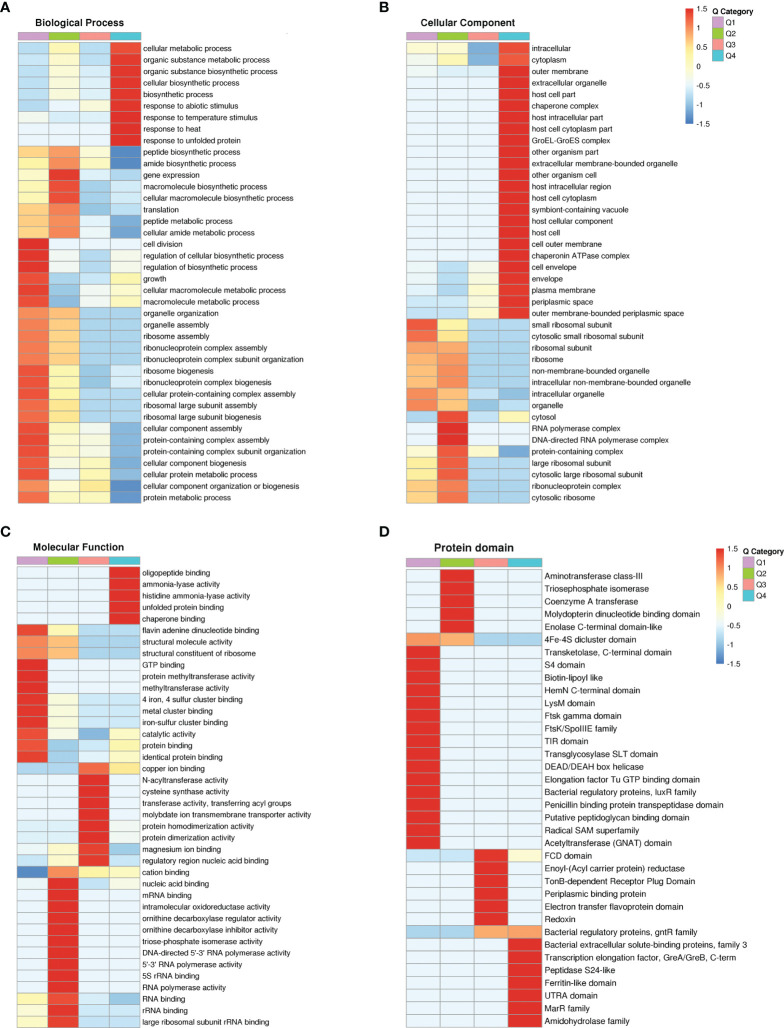
Functional cluster analysis of GO and protein domain of DEPs. **(A–C)** GO functional clustering analysis. **(D)** Protein domain functional clustering analysis.

KEGG pathway, GO and protein structural domains were then clustered to find the correlation between the functions of the differentially expressed proteins (**Supplementary Tables S6**–**S8**). Four sections (called Q1 to Q4) were grouped according to their differential expression ploidy (**Figure 3A**). For each Q group, GO classification, enrichment of KEGG and protein structural domains were performed separately, and cluster analysis was performed to find the correlations between the functions of the DEPs at different ploidy levels. KEGG enrichment analysis showed that significantly upregulated proteins (Q4) were enriched in five pathways, including galactose metabolism, ABC transporters, secondary bile acid biosynthesis, benzoate degradation, and aromatic compound degradation. The significantly down-regulate proteins (Q1) were enriched in four pathways, including ribosome, cell cycle-Caulobacter, peptidoglycan biosynthesis, and selenocompound metabolism. The upregulated and down-regulated DEPs were mainly concentrated in the ABC transporter and ribosome, respectively (**Figure 3B**). GO differential protein clustering analysis showed that the main differential proteins of A19 and A19BG were ribosome, ribosomal subunit, structural constituent of ribosome, structural molecule activity, organelle assembly and ribosome assembly. Down-regulated expression of a large number of ribosome-associated and intracellular life-activity-related proteins (**Figures 4A–C**). Clustering of differentially expressed proteins from different groups with functionally corresponding protein structural domains revealed upregulation of bacterial extracellular solute-binding proteins, MarR family, amidohydrolase family and bacterial regulatory proteins, gntR family et al. The structural domains of these proteins, such as transketolase, C-terminal domain, S4 domain, biotin-lipoyl like and HemN C-terminal domain were down-regulated in expression (**Figure 4D**).

### Main Outer Membrane Protein and Antigen Are Preserved in A19BG

In order to understand the antigen changes after 42°C -lysis, the differences in immunogenic antigens between A19 and A19BG were compared at the proteome level. Immunogenetic DEPs in A19BG are listed in **Table 1**. Bcsp31, Omp25, Omp10, Omp19, Omp28, and Omp2a were the main outer membrane proteins (OMPs), and DnaK, GroS, and GroL were the heat shock proteins (HSPs). These proteins have been evaluated as protective or immunoreactive antigens for subunit vaccines (22–24), and were expressed in both A19 and A19BG, but enriched in A19BG (**Table 1**). Moreover, LPS in A19BG was similar to that in A19, indicating that A19BG contained the most protective antigens of the parental strain A19. All these findings revealed that A19BG possessed enough immunogenetic antigens to elicit a protective immune response like A19.

**Table 1:** Immunogenic proteins co-occurring in A19 and A19BG.

Protein accession	Protein description	Gene name	A19BG/A19 Ratio
P0A3T3	31 kDa immunogenic protein	*Bcsp31*	2.9104
Q44664	25 kDa outer-membrane immunogenic protein	*omp25*	2.3528
Q2YIP8	Lipoprotein Omp10	*omp10*	9.4976
Q2YLR6	Outer membrane lipoprotein omp19	*omp19*	3.239
Q2YS14	Immunoreactive 28 kDa outer membrane protein	*omp28*	3.0466
Q44620	Porin Omp2a	*omp2a*	1.673
P15453	Superoxide dismutase [Cu-Zn]	*sodC*	2.615
Q2YQV2	Chaperone protein DnaK	*dnaK*	2.0825
Q2YIJ2	10 kDa chaperonin	*groS*	3.5838
P0CB35	60 kDa chaperonin	*groL*	3.2206

### Characteristic Antibody Profiles of A19BG Compared With A19 Immunisation

Proteome microarray was used to analyse antibody responses after vaccine immunisation to identify and compare the differences of immunogenic proteins. Antibody responses at 28 days post the immunisation were compared with before immunisation. The results showed that after immunisation with A19, antibodies to many proteins were detected, indicating that these proteins are immunogenic in A19. Of the top 20 immunogenic proteins, most of them are membrane proteins. The highly antigenic outer membrane proteins include VirB8, COML competence lipoprotein (BamD), peptidoglycan-associated lipoprotein, peptide ABC transporter substrate-binding protein, outer membrane lipoprotein, membrane fusion protein MTRC and porin family protein etc (**Table 2** and **Supplementary Table S9**). This also indicated that membrane proteins play a major role in the immune response induction for live *Brucella* A19 vaccines. Then, antibody profiles of A19BG were analysed and evaluated. Compared with that before immunisation, antibodies to a number of proteins were detected 28 days after immunisation with A19BG. Highly antigenic proteins included molecular chaperone GroEL, COML competence lipoprotein (BamD), peptidoglycan-associated lipoprotein, translocation protein TolB, outer membrane lipoprotein, outer membrane protein W, immunogenic protein bp28, outer membrane protein assembly factor BamA, porin family protein and VirB8 etc. However, antibodies to most ribosomal proteins and some intracellular proteins detected in the A19 group were absent in the A19BG group (**Table 2** and **Supplementary Table S10**). The above results showed that the humoral immune responses induced by A19 and A19BG were not similar.

**Table 2:** Top 20 immunogenic proteins after 28 days of A19 and A19BG immunisation.

A19			A19BG		
Antigen	Pre immunization (N = 5)	28 days post-immunization (N = 5)	Antigen	Pre immunization (N = 5)	28 days post-immunization (N = 5)
BMEII0032	0.223	3.04	BMEII1048	0.584	4.17
BMEI0587	–0.5929	2.45	BMEI0587	–0.459	4.52
BMEI0340	–0.6625	1.92	BMEI0141	0.247	2.21
BMEI1236	0.5283	1.79	BMEI0340	–0.466	2.05
BMEI0094	0.3343	1.51	BMEI1184	–0.440	2.66
BMEII0735	–0.0004	1.48	BMEI0339	0.281	1.76
BMEI1796	0.1411	0.91	BMEI0613	0.522	1.17
BMEI0668	0.0663	1.01	BMEI0135	0.09	2.83
BMEI0135	0.0239	2.49	BMEII0334	0.015	2.41
BMEI0892	0.1075	1.81	BMEI1829	0.084	0.75
BMEI1249	–0.0756	0.52	BMEI0454	0.541	1.46
BMEI0178	–0.4758	2.23	BMEI0536	0.041	1.82
BMEI0251	–0.2174	0.51	BMEI0830	–0.564	0.87
BMEI0376	0.2712	1.43	BMEI1249	0.119	0.69
BMEI1334	0.6047	1.21	BMEI1092	0.012	0.88
BMEI0324	0.219	2.25	BMEI0748	0.188	0.87
BMEI1536	0.2373	1.48	BMEII0032	0.368	1.06
BMEI0123	0.3078	1.1	BMEI1871	0.166	1.02
BMEI1646	0.0437	1.1	BMEI0123	0.308	1.33
BMEI1330	–0.3795	1.2	BMEI0673	0.25	0.78

The value represents the average reaction intensity.

### Signature Antigen Candidate for A19BG Immunisation

**Figure 5:**
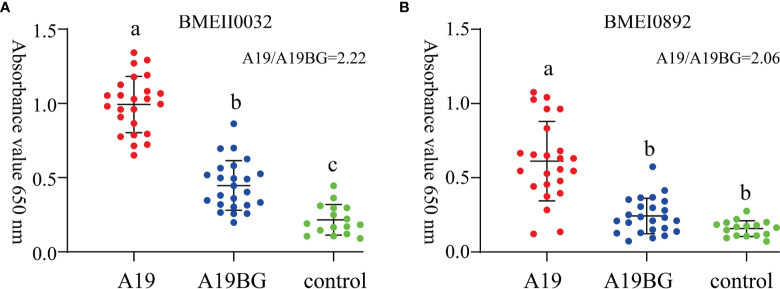
Immune response of BMEII0032 and BMEI0892 to A19 and A19BG immunisation sera. **(A)**, Immune response of BMEII0032 to A19 and A19BG immunisation sera. **(B)**, Immune response of BMEI0892 to A19 and A19BG immunisation sera. Means ± SEs, n =24, ELISA for *Brucella* negative sera as control, different letters indicate significant differences among treatments based on the least significant difference at P ≤ 0.01.

In order to investigate whether the immunogenic proteins of A19 and A19BG have the differential diagnostic ability, both comparative proteome and antibody profiles of the two strains were combined and analysed. The screening process of target antigens is shown in **Figure S1**. Proteins that are highly expressed and have high antibody levels in A19, but not detected in A19BG are ideal differential diagnosis antigens for A19BG. With this criteria, we screened the antigenic proteins, a number of proteins were identified (**Table 3**). To test its feasibility of use as differential diagnostic antigen, these proteins were expressed in *E. coli* and purified. Using these proteins as coating antigens, the antibody levels of the two clinical immune sera were detected by an indirect ELISA method. Finally, the results showed that the antibody levels of these two proteins, BMEII0032 and BMEI0892, in A19 immunization were significantly higher than those in A19BG immunization (**Figures 5A, B**), with both proteins having A19/A19BG values greater than 2. This suggests that the two antigens can distinguish A19 and A19BG immunization. In view of the high similarity between A19 and wild type strain, the two antigens have the potential to distinguish A19BG immunization from natural infection (25, 26).

**Table 3:** Differential IgG levels induced by different antigens 28 days after immunisation with A19 and A19BG.

Antigen	Description	A19 immunisation	A19BG immunisation
BMEII0032	VirB8	2.8164	0.6867
BMEI0178	hypothetical protein	2.7045	0.5428
BMEI0324	hypothetical protein	2.032	0.0887
BMEI0892	membrane fusion protein MTRC	1.7005	0.1638
BMEI0845	peptidyl-prolyl cis-trans isomerase D	1.6016	0.0198
BMEI0361	ABC transporter ATP-binding protein	1.5098	0.0305
BMEII0735	periplasmic oligopeptide-binding protein precursor	1.4821	0.6788
BMEI1236	exported proline-rich protein	1.2608	0.269
BMEI1630	acriflavin resistance protein A	1.2504	0.0372
BMEI1536	hypothetical protein	1.2467	0.0242
BMEI0094	vacuolar ATP synthase 16 KD proteolipid subunit	1.1788	0.0318
BMEI0376	hypothetical protein	1.1568	0.5256
BMEI1440	thiol:disulfide interchange protein DsbA	1.1364	0.0173
BMEI1646	acriflavin resistance protein E	1.0607	0.216
BMEI0668	calcium binding protein	0.9406	0.0484
BMEI0364	biopolymer transport EXBD protein	0.9361	0.0099
BMEII0988	copper-containing nitrite reductase precursor	0.8526	0.1439
BMEI0796	hypothetical protein	0.8299	0.3804
BMEI1079	lipoprotein NlpD	0.8008	0.5285
BMEI0228	LemA protein	0.7847	0.0305
BMEI1796	methyltransferase	0.7645	0.2427
BMEI1849	thiol:disulfide interchange protein CYCY precursor	0.7559	0.143
BMEI1521	acyl-CoA dehydrogenase	0.7522	0.0882
BMEI0251	ATP synthase F0F1 subunit beta	0.7321	0.055
BMEI1154	NADH dehydrogenase subunit E	0.709	0.4307
BMEI0613	protease Do	0.702	0.6481
BMEII0338	ABC transporter substrate-binding protein	0.6941	0.0796
BMEII0923	spermidine/putrescine-binding periplasmic protein	0.6802	0.0438
BMEI1487	colicin V production protein	0.6575	0.0784
BMEI0205	immunoglobulin-binding protein EIBE	0.6522	0.0188
BMEI1866	hypothetical protein	0.6452	0.3063
BMEI1439	chromosome segregation protein SMC2	0.6425	0.1025
BMEII0103	Leu/Ile/Val-binding protein precursor	0.6329	0.3559
BMEII0031	VirB7	0.3212	0.0012
BMEI0225	signal recognition particle subunit FFH/SRP54	0.1101	0.0018

The value represents the mean difference of reaction intensity between post-immunization and pre-immunization.

## Discussion

Although extensively applied for cattle brucellosis prevention, the *Brucella* A19 vaccine has limitations due to its residual virulence (27). In order to solve this problem, we developed the A19 bacterial ghost vaccine and proved its safety as well as effectiveness in guinea pigs and cattle (15). To reveal the safety and effectiveness mechanism of A19BG, this study conducted proteomic and antibody profile analysis of A19BG versus A19. Unlike A19, A19BG is an inactivated vaccine, and the total protein amount is fixed once inactivated. Therefore, the proteins in A19BG are directly related to immunisation efficiency. We chose highly sensitive proteomic techniques to study the protein components and immuno-protection mechanism A19BG. We conducted subcellular localisation analysis of DEPs and found that they were mainly located in the cytoplasm (**Figure 2B**), being consistent with the formation process of BGs, which are integral cell membrane- and periplasm-free of cell contents (28). There was also an attractive phenomenon on the cell membrane: the number of DEPs in the cytoplasmic membrane was much higher than that in the outer membrane, with mainly down-regulated DEPs (16%) found in the cytoplasmic membrane and DEPs found in the outer membrane were all upregulated ones (**Figure 2C**). These results indicated that BGs do not retain all the membrane components, strengthening the traditional concept of BG components. So far, there is no detailed proteomic analysis on the distinct components of BGs. It was speculated that the damage of the inner membrane occurred in the lysis tunnel because the inner membrane was incomplete in the lysis tunnel of E-lysed *E. coli* (29). However, the outer membrane was also damaged because of the fusing between the two membranes in the lysis tunnel. We also speculated that the perforin expressed by the E-lysis gene could destabilise the inner protein components as the inner membrane is the carrier of many biological processes, such as biosynthesis, transport, and DNA anchoring (30). When cytoplasmic components are lost, the inner membrane is generally disturbed. This conjecture was confirmed by KEGG pathway analysis of cytoplasmic DEPs (**Table S6**). The KEGG pathways of cytoplasmic DEPs were down regulated, including substance transport and homeostasis maintenance, such as bacterial secretion system, ABC transporters, two-component system, and quorum sensing. In addition, it also included the synthesis and metabolism of substances and energy production, such as peptidoglycan biosynthesis, metabolic pathways, citrate cycle (TCA cycle), and oxidative phosphorylation (**Table S6**). Therefore, the complete membrane structure of BGs mentioned in the literature may not be correct, which may have large-scale damage at the protein level. Such subtle damage cannot be verified by electron microscopy. Future studies can continue to use omics to analyse the structure of different BGs.

The first concern of BG vaccine preparation is the destruction of antigen epitopes or immunogenic antigens. As previously mentioned, we have demonstrated that A19BG was as safe and protective in guinea pigs as A19. Moreover, there are many BG vaccines of other bacteria that have been well-studied and put into use, such as *Actinobacillus pleuropneumoniae* (31), *Pasteurella* (32), *Escherichia coli* O157:H7 (33), *Salmonella enteritidis* (34), *Salmonella typhimurium* (35), *Brucella suis* (13), and S2308 (14), all of which could produce effective humoral and cellular immunity. Moreover, *Actinobacillus pleuropneumoniae* BGs could effectively prevent lung colonisation and immune carriers. However, it has not been reported whether BGs really fully preserve all proteins related to immune protection.

Previous studies have shown that protective antigens such as Cu-Zn, BP26, SOD, bcsp31,GroEL, GroES, DnaK and outer membrane protein family are involved in the immune response or protective immunity induction for *Brucella* (36). In the present study, by using antibody profiles, these proteins were also found to be involved in the induction of immune response to A19 and A19BG. Immunogenic proteins were identified in both the whole cell and membrane proteins of *Brucella*. A total of 61 proteins were determined to be highly immunogenic, among which elongation factor G, F0F1 ATP, synthase subunit beta and OMP1, were identified to be immunogenic for the first time (37). Some proteins located in the cytoplasm, such as 50s ribosomal protein L10 and ribosomal protein L7/L12, showed significantly reduced protein contents and antibody response in A19BG and A19BG vaccination. Interestingly, many protective antigens located on membranes, including OMP31, OMP25, OMP19 and OMP16, remains largely unchanged both in A19BG proteome and antibody profiles. This also validates that A19BG have retained main protective antigens that are essential for protection induction (38–40).

Other noticeable proteins were non-membranous antigenic proteins, such as SodC and HSP chaperones (DnaK, GroS, and GroL) (**Table 2**); these proteins were retained in A19BG and did not undergo a significant reduction in content. SodC exists in the periplasm, contributes to the antioxidant defence system, and protects bacteria from the toxic effects of reactive oxygen intermediates (41). DNA vaccine encoding SOD Cu/Zn superoxide dismutase- IL-2 fusion protein, induced IgG2a and TNF-α in mice, leading to effective protection against *B. abortus* 2308 strain similar to *B. abortus* RB51 vaccine (42). HSPs are located in the cytoplasm and their main function is to maintain protein folding and homeostasis under a large variety of stress conditions (43). Furthermore, these proteins could produce specific antibodies in serum after RB51 immunisation and *B. suis* infection, indicating that they have good immunogenicity (44). HSP chaperones are not only immunogenic but also act as immunomodulators. DnaK co-immunised with *Brucella* OMP22 modulated the immune response, specifically the CMI (45). Therefore, the presence of these non-membrane proteins further enhances the immunoprotective effect of A19BG as a vaccine.

Besides, cell membrane proteins are also delicate to elicit a host immune response. More importantly, membrane proteins are the first contact in the interaction of bacteria and host cells (46). We found that compared with A19, the expression level of Omp25, Omp10, Omp19, Omp28, Omp2a and BCSP31 of A19BG were significantly no reduction, retains comparable cell membrane composition to A19 (47). Because these proteins have good immunogenicity, A19BG can induce a strong immune response after booster immunization (**Table 1**). In view of the better immunogenicity of outer membrane proteins, many candidate subunit vaccines for *Brucella* are outer membrane proteins. The recombinant Omp25 produced Th1 and Th2 immune responses in BALB/c mice with comparable protection to the S19 vaccine (48, 49). Similarly, immunising BALB/c mice with uncertified Omp19 stimulated the production of antigen-specific CD4+ or CD8+T cells with a similar protective effect on S19 (50). In order to better provoke the immune response, simultaneous recombination of several immunogenic proteins can make up for the deficiency of a single subunit vaccine, such as the combination of Omp16, Omp19, Omp28, and L7/L12, or SOD, L7/L12 and Bcsp31 (51, 52). In contrast with single or multiple protein(s) recombinant subunit vaccines, BG has a more prominent advantage in terms of the number of ingredients and retains the natural structure of the protein. In general, A19BG retains the important immunogenic antigen components of the parent strain, such as membrane protein, LPS, lipoprotein, etc., in which LPS also has strong immune stimulating properties. After inoculating animals, A19BG can effectively induce immune response, and the protective effect provided by A19BG vaccination is similar to that of A19. A19BG vaccine does not contain live bacteria, which can avoid accidental infection of operators during vaccination.

The antibody profiling studies carried out in this study have confirmed the proteomic analysis and have identified many new immunoreactive proteins. Some proteins with antibody response were detected in A19BG immune antibody spectrum, but not at proteome level, such as 50S ribosomal protein L7/L12. We analyzed these as a non-specific protein of Brucella, which has cross reaction with other gram-negative bacteria. Therefore, the target for *Brucella* antibody diagnosis needs to be highly specific, free from other Gram-negative bacteria, and able to address the issue of differentiating between vaccine immunity and natural infection. The diagnosis of brucellosis in the last decades has been based mainly on anti-smooth LPS (S-LPS) antibodies (53, 54). However, anti-S-LPS antibodies have cross-reactivity with other Gram-negative bacteria. Therefore, identifying specific immunogenic proteins to develop LPS-free and protein-based diagnostics has become a research priority (55, 56). In this research, antibody profile identified a large number of antigens from which the A19 and A19BG immune sera could be distinguished, and these antigens were not only immunogenic but also had the potential to discriminate between the two vaccines. In this study, we compared the immune response levels of BMEII0032 and BMEI0892 in A19 and A19BG immunized bovine serum using indirect ELISA. The results showed that these two antigens could well differentiate between A19 and A19BG immunisation and have a good differential diagnosis. It is further proposed that screening of new target proteins for differential detection of *Brucella* BGs vaccine.

## Conclusion

A19BG vaccine possess the main immunogenetic antigens of A19 but with non-pathogenicity, which were safer in prevention of brucellosis comparing to live attenuated vaccines. BMEII0032 and BMEI0892 could be potential antigens for differentiating diagnosis from A19 and A19BG immune sera. The study provided the basis for improving the current vaccine and developing differentiating diagnosis methods of Bovine brucellosis. Further evaluation of the A19BG vaccine and differentiating diagnosis method are desired to control and eliminate animal brucellosis in China.

## Data Availability Statement

The datasets presented in this study can be found in online repositories. The names of the repository/repositories and accession number(s) can be found below: http://www.proteomexchange.org/, accession ID: PXD031623.

## Ethics Statement

The animal study was reviewed and approved by the Committee on Ethics and Welfare of Experimental Animals of Tecon biological Co., Ltd.

## Author Contributions

Z-LC, SH, and B-SL conceived and designed the experiments. M-ZL, H-LZ, and Q-LY performed the experiments. C-YH, J-HY, and Y-BY analysed the data and drafted the manuscript. All authors contributed to the article and approved the submitted version.

## Funding

This work was supported by the NSFC International (Regional) Cooperation and Exchange Program [grant number 31961143024], State Key Program of the National Natural Science Foundation of China [U1808202], Key Program of Inner Mongolia [grant number 2019ZD006], National Key Research and Development Program Projects [grant numbers 2017YFD0500901, 2017YFD0500305, 2016YFC1200100], and the National Key Program for Infectious Disease of China [grant number 2018ZX10101002-002].

## Conflict of Interest

Authors C-YH, M-ZL, H-LZ, Q-LY, and SH are employed by Tecon Biological Co., Ltd.

The remaining authors declare that the research was conducted without any commercial or financial relationships that could be construed as a potential conflict of interest.

## Publisher’s Note

All claims expressed in this article are solely those of the authors and do not necessarily represent those of their affiliated organizations, or those of the publisher, the editors and the reviewers. Any product that may be evaluated in this article, or claim that may be made by its manufacturer, is not guaranteed or endorsed by the publisher.

## Supplementary Material

Supplementary Figure 1 Initial analysis of date and protein identification. **(A)** MS/MS spectrum database search analysis summary. **(B)** The number of up- and down- regulated DEPs. **(C, D)** Identified peptide length distribution **(C)** and protein mass distribution **(D–F)**, principal component analysis **(E)** and Pearson’s correlation coefficient **(F)** of groups.

Supplementary Figure 2 The screening process of differential diagnosis antigens for A19BG immunization. First, whole proteins were extracted from A19 and A19BG, digested with trypsin, followed by LC–MS/MS analysis. The protein expression profiles of A19 and A19BG were compared and analyzed. Subsequently, proteins that are highly expressed and have high antibody levels in A19, but not detected in A19BG were screened. Finally, the antigen with identification potential was identified by indirect ELISA.

Supplementary Table 1 Identified results of mass spectrometry.

Supplementary Table 2 Differentially expressed proteins in the groups of A19BG and A19.

Supplementary Table 3 COG analysis for DEPs in the groups of A19BG and A19.

Supplementary Table 4 Subcellular location analysis for DEPs in the groups of A19BG and A19.

Supplementary Table 5 GO enrichment analysis in A19 and A19BG.

Supplementary Table 6 Cluster analysis of KEGG enrichment.

Supplementary Table 7 Cluster analysis of GO enrichment.

Supplementary Table 8 Cluster analysis of protein domain enrichment.

Supplementary Table 9 Antigenic protein in A19 immunization.

Supplementary Table 10 Antigenic protein in A19BG immunization.
