# Supplementary figures and images for: Proteomic and Antibody Profiles Reveal Antigenic Composition and Signatures of Bacterial Ghost Vaccine of Brucella abortus A19
Source: Front Immunol. 2022 Apr 22;13:874871. doi: 10.3389/fimmu.2022.874871 (PMC9074784; doi:10.3389/fimmu.2022.874871)

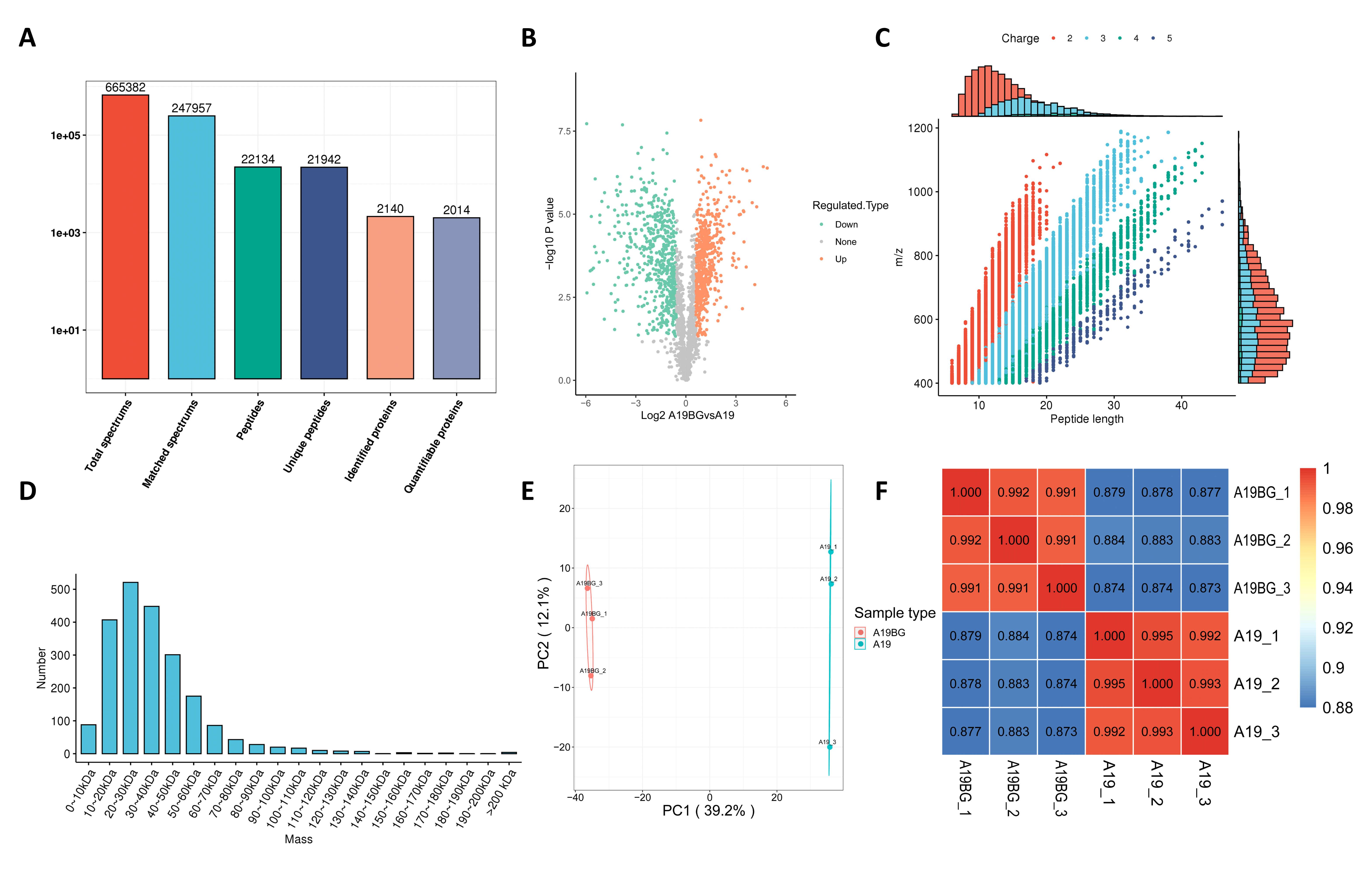

Supplement: Supplementary Figure 1 — Initial analysis of date and protein identification. (A) MS/MS spectrum database search analysis summary. (B) The number of up- and down- regulated DEPs. (C, D) Identified peptide length distribution (C) and protein mass distribution (D–F), principal component analysis (E) and Pearson’s correlation coefficient (F) of groups. [file Image_1.tif]

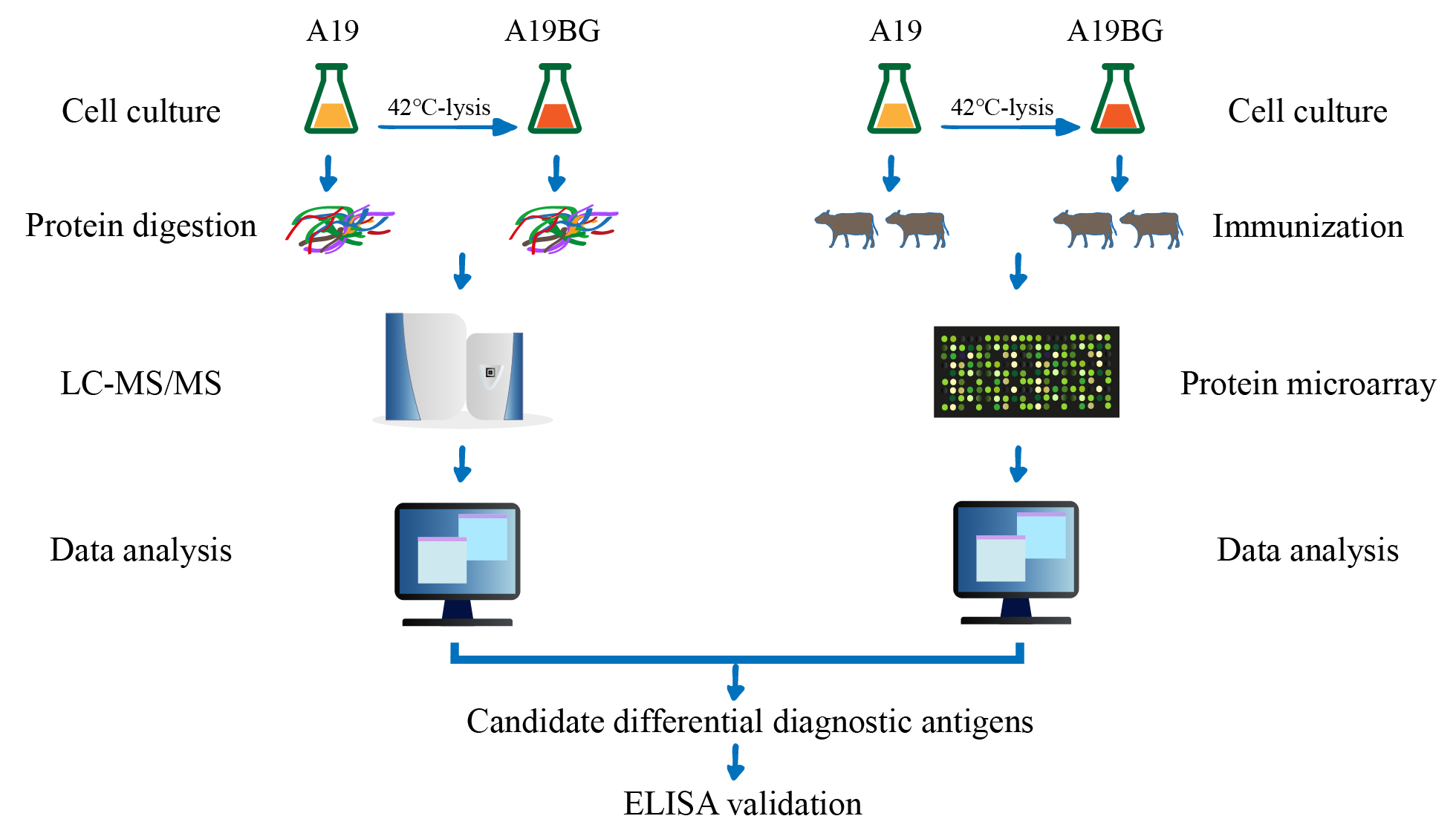

Supplement: Supplementary Figure 2 — The screening process of differential diagnosis antigens for A19BG immunization. First, whole proteins were extracted from A19 and A19BG, digested with trypsin, followed by LC–MS/MS analysis. The protein expression profiles of A19 and A19BG were compared and analyzed. Subsequently, proteins that are highly expressed and have high antibody levels in A19, but not detected in A19BG were screened. Finally, the antigen with identification potential was identified by indirect ELISA. [file Image_2.jpeg]
